# Dr. Burrows—The Injustice Done Him

**Published:** 1830-07-01

**Authors:** 


### Dr. Burrows.

We have perused the letter which Dr. B. has addressed to Sir Henry Halford, on the subject of the late trial, and its contents confirmed us in the opinion we had previously formed, that Dr. Burrows was a wronged, and what is worse, a much injured practitioner. It is difficult to account for the rancour and determined hostility with which Dr. Burrows has been pursued, from the case of Anderton, down to the present time. It was such as might naturally enough lead to a conviction, in his own mind, that a conspiracy existed against him. But we have no doubt that all this hostility sprung from personal enmity. Thus, for example, there is now little question that the recent severe animadversions in the Quarterly Review were penned by the late Dr. Gooch, when on his death-bed! Neither can it be much doubted that this bitterness of spirit, on the part of the late talented and amiable physician grew out of some literary altercations between him and Dr. Burrows. The tenor of the review is clearly not that of an unbiassed and unprejudiced critic. But, "de mortuis nil nisi bonum." Dr. Burrows has still more reason to complain of Mr. Brougham, whose language and observations were equally unjust and unwarrantable. It is lamentable, indeed, that a respectable medical man's reputation may be destroyed, and his family ruined by the unbridled and unmeasured language of an advocate, contending for mastery in a law-suit, without any regard to the truth or justice of the case.

Dr. Burrows' conduct has been so completely exculpated already, in the minds of all medical men, that we need not go over the grounds of his letter to Sir Henry Halford. But the mischief is done—and the public prejudice is excited through innumerable ramifications where the justification cannot permeate. We conceive it to be the duty of the medical profession to remedy, as far as possible, the evil, and protect a respectable and deeplyinjured member from ruin, by counteracting the impression that has gone forth, and, by patronising the individual, till the pressure of the calamity shall have been removed by time. We hope this will be borne in mind by our brethren, and we do not exhort them to do that which we shall fail to perform ourselves, whenever the opportunity occurs.

